# Application of Mixed Shell Powder as Modifier and Filler in Asphalt Mixture

**DOI:** 10.3390/ma19101968

**Published:** 2026-05-10

**Authors:** Chunyan Wang, Yafan Yang, Fangyuan Gong, Xuejiao Cheng, Bohan Ma

**Affiliations:** 1 School of Civil and Transportation, Henan Vocational University of Science Technology, No. 6 East Wenchang Avenue, Zhoukou 466000, China; 2 School of Civil and Transportation Engineering, Hebei University of Technology, 5340 Xiping Road, Beichen District, Tianjin 300401, China

**Keywords:** pavement materials, shell waste, modified asphalt, road performance, tropical island pavement

## Abstract

The rapid development of tropical island tourism has put forward a higher demand for asphalt pavement construction on the island. However, the asphalt pavement engineering in the offshore area is generally faced with high material transportation costs. Additionally, challenges such as high-temperature climate and heavy-load traffic may lead to permanent pavement deformation. As a typical marine solid waste, shells have high calcium carbonate content and porous structures, which have the potential advantage of modified asphalt. In this study, mixed shell powder was used as a modified material, and 70 # base asphalt and SBS-modified asphalt were mixed, respectively. The effect of asphalt modification was analyzed by basic performance tests and high-temperature rheological tests. An asphalt mixture was prepared by replacing limestone powder with mixed shell powder in equal volume, and its road performance was systematically tested. The modification mechanism was revealed by means of a microscopic test. The results show that the recommended content of mixed shell powder in SBS-modified asphalt is 9%, and 50–100% mixed shell powder can be used to replace mineral filler in base asphalt and single SBS modified asphalt mixture. This study provides effective technical support for the utilization of shell solid waste in offshore areas and the optimization of asphalt pavement performance.

## 1. Introduction

The road transportation industry has become an important support for the balanced development of the regional economy and human resources. Improving the construction of transportation infrastructure on the island has become an important way to enhance the competitiveness of island tourism, which not only helps to fully develop the ecological resources of tropical islands and reefs but also promotes their rational utilization [1,2]. Asphalt pavement is widely used in island highway construction because of its good flatness, low noise, and short construction period [3]. For asphalt road construction in offshore areas, all kinds of materials need to be transported by barge, which will produce a certain transportation cost. In view of the pressure brought by this cost, it is particularly important to explore the locally available resources.

As common marine ecological resources, shells include scallop shells, oyster shells, snail shells, mussel shells, clam shells, and so on. The primary components of typical shells are more than 95% calcium carbonate, along with small amounts of organic matter. Studies have shown that properly treated shells can be used as partial substitutes for traditional building materials, contributing to the stabilization of unstable soil and partially replacing non-renewable mineral resources such as cement and aggregates, thereby offering significant economic and environmental benefits [4,5,6]. Kamrul H et al. [7] and Yang B et al. [8] observed that the use of shells as a cement substitute in concrete may hold potential for producing sustainable green building materials and effectively reducing environmental pollution. The optimal substitution ratio is typically between 5% and 20%. Her S et al. [9] confirmed that, in clinker partially replaced by cement produced using shell powder as a raw material, the formation of the anhydrous phase—as well as the type and morphology of the hydration products—remains consistent with that of ordinary Portland cement. Yusof M et al. [10] found that concrete containing 15% clamshell powder exhibited properties with the difference less than 5% to the reference concrete without clamshell powder.

In asphalt-specific studies, the porous structure of shell powder holds potential for improving the high-temperature rheological properties of asphalt. To avoid adverse effects on ductility, the typical addition rate is generally kept below 15% [11]. Guo et al. [12] and Wang et al. [13] have shown that only physical mixing of shell powder and asphalt can improve the consistency, hardness, and high-temperature rheological properties of asphalt but weaken the low-temperature performance. Chen Y et al. [14] found that the addition of an appropriate amount of scallop shell powder can improve the thermal stability and permanent deformation resistance of asphalt by up to 20%. Key components and existing application research indicate that using waste shell powder as a filler in asphalt mixtures enables the utilization of solid waste while also improving certain mechanical properties of the asphalt mixture. Studies typically involve high-proportion or complete substitution [15,16,17]. Ruiz Gaby et al. [18] found that waste seashell powder as a substitute for fine aggregate in asphalt mixtures significantly increases the tensile strength ratio by 22%, despite a corresponding 4.6% increase in material costs. Alabani M et al. [19] indicated that shells as hot-mix asphalt fillers can improve material fatigue life and resistance to permanent deformation across varying temperatures. Existing studies have primarily focused on the single-application use of specific types of shell powder. However, waste shells typically consist of various types; research into the comprehensive application of mixed shell powder in asphalt mixtures holds practical significance.

To investigate the effects of mixed shell powder applied as a modifier and filler substitute in asphalt mixtures and to determine the recommended dosage combined with a multi-index comprehensive evaluation, this study aims to provide theoretical and technical support for the application of shell solid waste in tropical island pavement engineering. Based on the indices of penetration, softening point, ductility, and 135 °C Brookfield rotational viscosity, the influence of mixed shell powder as a modifier on the basic properties of asphalt was systematically analyzed. The high-temperature rheological properties of shell-powder-modified asphalt were further analyzed by a temperature scanning test and multiple stress creep recovery tests. The road performance of asphalt mixtures prepared with mixed shell powder as a substitute for limestone filler was evaluated. With the help of scanning electron microscopy and Fourier transform infrared spectroscopy, the modification mechanism was explored from a microscopic point of view.

## 2. Materials and Methods

### 2.1. Materials

In this study, Shell 70 # road asphalt and SBS-modified asphalt were selected as experimental materials. The coarse and fine aggregates and fillers used in this study are all limestone materials. The particle size of limestone powder is 0.045~0.075 mm, the apparent density is 2.63 g/cm^3^, and there is no agglomerate in appearance. The performance indicators of the above materials meet the technical requirements of ‘Technical Specification for Highway Asphalt Pavement Construction’ (JTG F40-2004) [20]. The mixed shell powder was produced by Shandong Futian Zhengda Biological Technology Co., Ltd. (Binzhou, China) with a calcium carbonate content of up to 95%, a particle size of 0.045~0.075 mm, and an apparent density of 2.27 g/cm^3^. It is similar to the performance of limestone powder. Therefore, it can be used as a substitute for limestone powder in an asphalt mixture. The main technical indicators of materials are shown in Table 1.

### 2.2. Preparation Method

**Figure 1:**
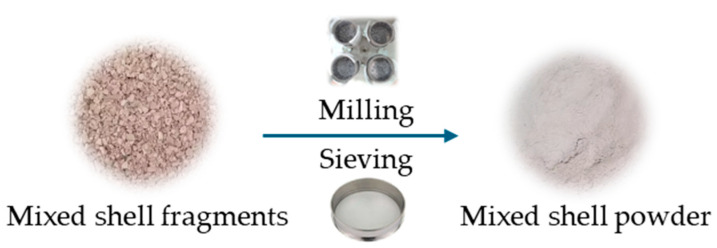
Preparation process of mixed shell powder.

The mixed shell fragments selected in this experiment were high-calcium. Firstly, the collected mixed shell fragments were thoroughly washed and then dried in an oven at 105 ± 5 °C until a constant weight was reached. Then, the mixed shell fragments and stainless steel balls were placed in the QM-3SP2 planetary ball mill for grinding (500 rpm) (Nanjing Nanda Instrument Co., Ltd., Nanjing, China). Finally, the standard sieve with a pore size of 0.075 mm was used for screening. The preparation process of mixed shell powder is shown in Figure 1.

The preparation process of mixed shell powder-modified asphalt is designed as follows. Firstly, the asphalt is heated to 160 °C. In the process of mixing the powder into the asphalt, the powder was added to the asphalt 3 to 4 times, and the mixture was stirred at a low speed with a stirring rod and gradually heated to 180 ° C until no free powder that was not bound to the asphalt was observed. Then, the FJ300-SH high-speed dispersion homogenizer (Shanghai Huxi Industrial Co., Ltd., Shanghai, China) was used to shear for 45 min at a speed of 3000 r/min so that the powder was uniformly dispersed in the asphalt to obtain a mixed shell powder-modified asphalt.

**Figure 2:**
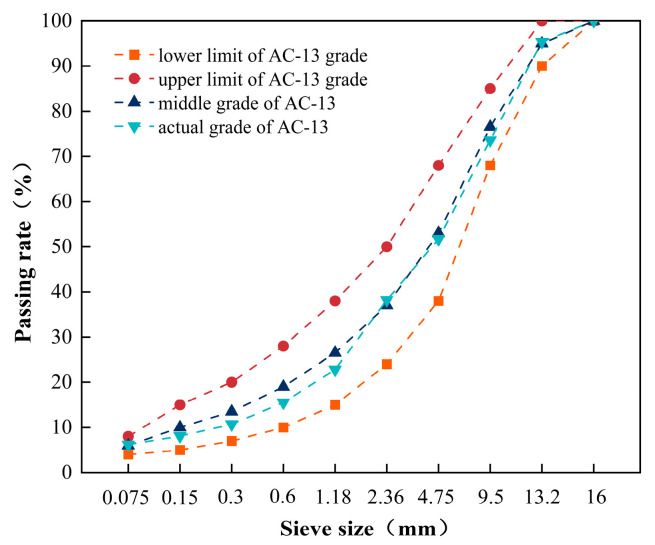
Gradation of asphalt mixture (AC-13).

Considering the complexity of shell species in offshore areas and the relatively limited number of shells of a single species, it is difficult to classify and recover them. Therefore, compared with a single type of shell powder, the mixed shell powder has a wider source and is more advantageous in terms of the convenience of collection. Therefore, this research plans to select different contents (3%, 6%, 9%, 12%, 15%, and 18%) of mixed shell powder to prepare mixed shell powder-modified asphalt by adding 70 # base asphalt and SBS-modified asphalt, respectively. The mixed shell powder content is defined as a percentage by weight of the asphalt mass (external addition method). Then, three kinds of mixed shell powder with substitution amounts of 0%, 50%, and 100% were used to replace limestone powder in equal volume to prepare SBS-modified asphalt mixture, mixed shell powder/SBS-composite-modified asphalt mixture, and base asphalt mixture. Continuous gradation AC-13 is adopted in this study, and the specific design gradation and gradation curve of mineral aggregate are shown in Figure 2. The oil–stone ratio was determined by the Marshall test method. Five Marshall specimens with different oil–stone ratios were selected at an interval of 0.3%. The relative density and theoretical density of gross volume corresponding to different oil-stone ratio specimens were measured. The three-volume parameters of porosity, mineral aggregate porosity, and asphalt saturation were calculated, and the stability and flow value were measured by the Marshall test. According to the OAC method in JTG F40-2004 [20], the optimum asphalt–aggregate ratio is 4.9%.

### 2.3. Test Methods

#### 2.3.1. Basic Performance Tests

In this experiment, according to ‘Highway Engineering Asphalt and Asphalt Mixture Test Procedures’ (JTG 3410-2025 T 0604, T 0605, T 0606, and T 0625) [21], the SYD-2801E penetration tester was used to determine the penetration depth of a 100 g standard needle through the asphalt sample heated at 25 °C within 5 s. The difference between the results of three parallel tests was less than 0.2 mm. In the ductility test, the asphalt in the standard mold was stretched to fracture at a speed of 5 cm/min at 10 °C and 5 °C with the help of the SY-1.5B constant temperature digital display asphalt elongation tester (Cangzhou Kexing Instrument Equipment Co., Ltd., Cangzhou, China), and the length at fracture was measured. The samples were subjected to three parallel tests, with a relative allowable error of less than 20%. The softening point test used the SYD-2806E automatic asphalt softening point tester (Cangzhou Kexing Instrument Equipment Co., Ltd., Cangzhou, China) to measure the heating temperature of the 3.5 g standard steel ball from the metal ring filled with the asphalt sample to the contact with the lower bottom surface. Two parallel tests met the 1 °C tolerance limit. The rotational viscosity test method was used with the No.27 rotor, and the temperature was set to 135 °C. The average of three consecutive readings was taken as the test result; the relative allowable error for the repeatability test was 3.5%.

#### 2.3.2. Dynamic Shear Rheological Tests

In order to evaluate the high-temperature performance and viscoelasticity of asphalt, an Anton Paar MCR 102 dynamic shear rheometer was used to carry out the test (JTG 3410-2025 T 0628 and T 0647) [21]. The temperature scanning test was carried out under the strain control mode (strain 12%, frequency 10 rad/s). The temperature was increased from 46 °C to 88 °C at 6 °C intervals, and the dynamic shear modulus G*, phase angle δ, and rutting factor G*/sinδ were obtained. When G*/sinδ < 1, the failure temperature was determined. When the multiple stress creep recovery test is carried out, 52 °C, 58 °C, and 64 °C are selected as the test temperature; 0.1 kPa and 3.2 kPa are the two test stresses. The asphalt is subjected to cyclic loading for 20 cycles at a stress level of 0.1 kPa and cyclic loading for 10 cycles at a stress level of 3.2 kPa. Each cycle consists of a 1 s loading time and a 9 s unloading recovery time. The strain recovery rate R and the unrecoverable creep compliance J_nr_ are the test indicators. There were two parallel specimens in the above tests. The relative allowable errors for the repeatability tests of G*/sinδ, R_0.1_, R_3.2_, J_nr0.1_, and J_nr3.2_ are 5.2%, 4.2%, 4.7%, 26.5%, and 26.8%, respectively.

#### 2.3.3. Asphalt Mixture Performance Test

Based on the JTG 3410-2025 T 0719, T 0715, and T 0729 [21], the size of the asphalt mixture plate specimen used in the rutting test was: length of 300 mm × width of 300 mm × height of 35 mm. The specimens were placed in a constant temperature of 60 ± 1 °C for more than 5 h. The wheel pressure was set at 0.7 MPa, and the running time is 1 h. The variation coefficient for repeatability tests of dynamic stability between two parallel specimens was less than or equal to 20%. The test temperature of the low-temperature bending test was −10 ± 0.5 °C, and the loading rate was 50 mm/min. The rut plate was cut into a prism beam used in the test of length of 250 mm × width of 30 mm × height of 35 mm. The maximum load and mid-span deflection of the specimen were obtained, and the maximum bending strain ε_B_ and bending strength R_B_ of the specimen were calculated. The measured ε_B_ of three parallel specimens met the allowable error of 400 με for repeatability testing. The standard Marshall specimens were used for the freeze–thaw splitting test. The freezing temperature was −18 °C, and the freezing time was 16 h. Then, the frozen specimen is transferred to a constant-temperature water tank with a temperature of 60 °C for 24 h. Finally, the frozen specimen and the remaining specimen are placed in a constant-temperature water tank at 25 °C for 2 h to prepare for the subsequent splitting test. The measured tensile strength ratio (TSR) of four parallel specimens met the relative allowable error of less than 15% for repeatability testing.

#### 2.3.4. Microscopic Characterization Tests

The TENSOR 27 Fourier infrared spectrometer was used to carry out experimental analysis. The test range is set to be a 4000–400 cm^−1^ wave number interval, the resolution is 0.5 cm^−1^, and the number of scans is 32 times to ensure the accuracy and stability of the spectral signal. A JEOL series scanning electron microscope (JEOL Ltd., Tokyo, Japan) was used to observe the distribution state, microstructure characteristics, and interface bonding of mixed shell powder in asphalt. The 100-fold, 500-fold, 3000-fold, and 5000-fold magnifications were selected to observe the overall structure and dispersion details of the shell powder.

## 3. Results and Discussion

### 3.1. Performance Analysis of Mixed Shell Powder-Modified Asphalt

#### 3.1.1. Basic Performance Tests

**Figure 3:**
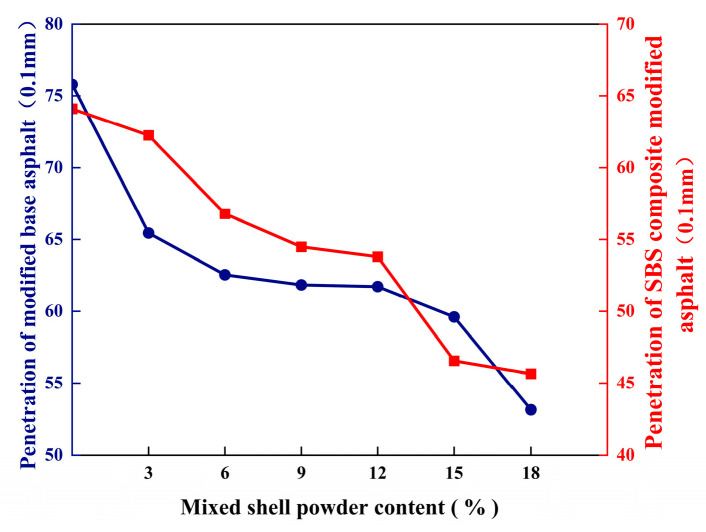
Penetration of asphalt with different proportions of mixed shell powder.

Penetration can reflect the hardness of asphalt at the standard test temperature. The effect of different mixed shell powder contents on the penetration of base asphalt and SBS-modified asphalt is shown in Figure 3. The mixed shell powder modified asphalt was prepared by adding different contents (3%, 6%, 9%, 12%, 15%, and 18%) of mixed shell powder into 70 # base asphalt and SBS-modified asphalt. The penetration of base asphalt decreased by 13.7%, 17.5%, 18.4%, 18.6%, 21.4%, and 29.8%, respectively, and the penetration of SBS-modified asphalt decreased by 2.9%, 11.4%, 15.0%, 16.1%, 27.4%, and 28.8%, respectively. It is because the mixed shell powder fills the internal structure of the asphalt and increases the consistency and stiffness of the asphalt. Therefore, as the content of the mixed shell powder increases, the penetration of the asphalt gradually decreases.

**Figure 4:**
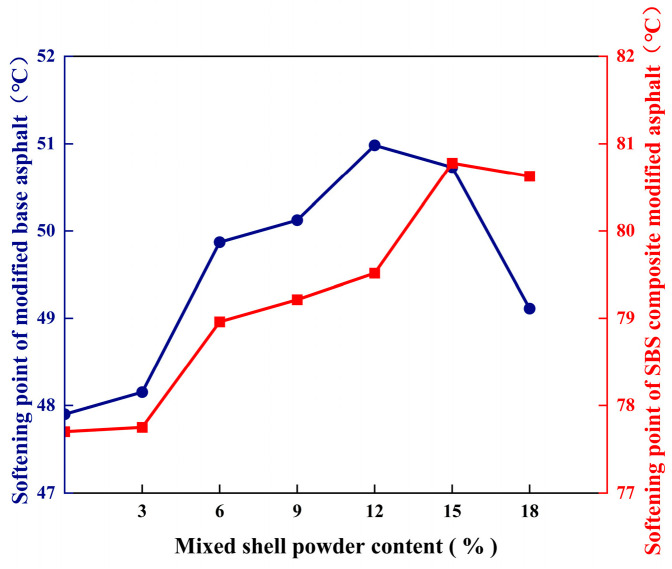
Softening point of asphalt with different proportions of mixed shell powder.

The softening point refers to the softening and sagging temperature of the asphalt specimen after heating, which can evaluate the high-temperature stability of the asphalt. Figure 4 shows the softening point of mixed shell powder modified asphalt and mixed shell powder/SBS composite modified asphalt. The softening point of modified asphalt with different mixed shell powder content is higher than that of the original asphalt, and the softening point increases with the increase in the content. When the content reaches a certain value, the softening point decreases. When the content of mixed shell powder in base asphalt is 12%, the softening point is the highest, which is 51.0 °C. When the content of SBS-modified asphalt is 15%, the softening point is the highest, which is 80.8 °C.

**Figure 5:**
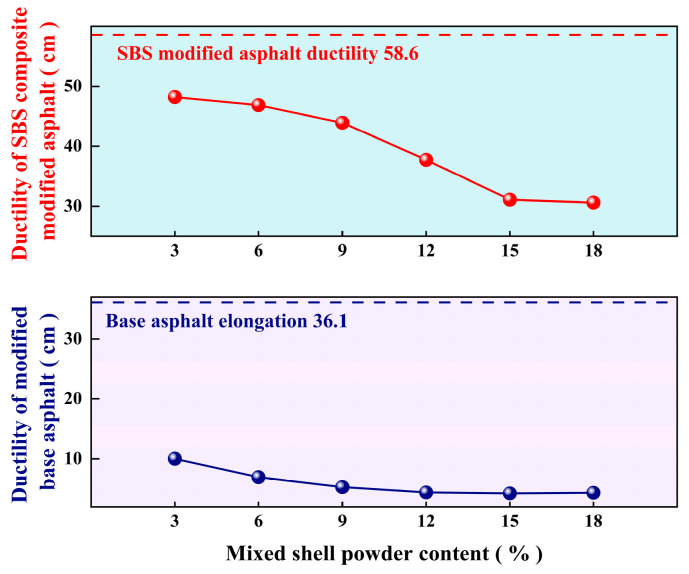
Ductility of asphalt with different proportions of mixed shell powder.

Figure 5 shows the effect of mixed shell powder on the ductility of two kinds of asphalt: after mixing with mixed shell powder, the ductility of base asphalt is significantly reduced and cannot meet the requirements of JTG F40-2004 [20]. For SBS-modified asphalt, when the content of mixed shell powder exceeds 9%, the ductility also decreases significantly. The main factors causing the above phenomenon include, first of all, the mixed shell powder being composed of a variety of shells, and the content of organic matter (such as shell matrix protein, and polysaccharide, etc.) contained in different shells being different, resulting in the instability of its compatibility with asphalt, affecting the interface bonding effect. Secondly, the mixed shell powder may retain impurities such as barnacles, introduce non-calcium carbonate components, and destroy the uniformity of the material. Furthermore, the microstructure and pore characteristics of different kinds of shells are different, and the specific surface area and surface energy between shell powder particles are also different, which makes it easy to form aggregates due to electrostatic adsorption or surface energy difference. Finally, due to the poor ductility of the base asphalt itself, the rigid particles of the shell powder will destroy its structural continuity, making the asphalt more brittle in the low-temperature environment.

**Figure 6:**
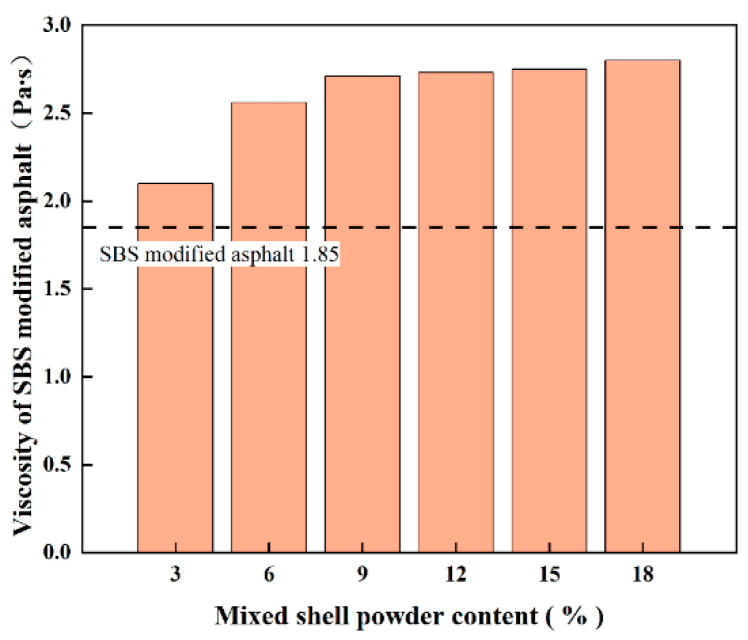
Viscosity of SBS-modified asphalt with different dosages of mixed shell powder.

Generally speaking, the higher the viscosity of asphalt, the worse its fluidity and the stronger its ability to resist deformation, but it will also increase the difficulty of construction. On the contrary, low-viscosity asphalt has good fluidity and more convenient construction, but there may be a risk of poor adhesion to aggregate. In view of the fact that the ductility of mixed shell powder-modified base asphalt can not meet the relevant requirements, this study only explores the effect of mixed shell powder on the viscosity of SBS-modified asphalt. Figure 6 shows the influence of different amounts of mixed shell powder on the rotational viscosity of SBS-modified asphalt at 135 °C. The test results show that with the gradual increase in the content of mixed shell powder, the rotational viscosity of SBS-modified asphalt shows a continuous upward trend, so the recommended content is 9%.

#### 3.1.2. Dynamic Shear Rheological Tests

From the above basic performance tests, it can be obtained that the mixed shell powder as a modifier improves the softening point of the base asphalt and SBS-modified asphalt, reduces its penetration and ductility, and when the mixed shell powder content is 3%, the ductility of the base asphalt does not meet the specification requirements. Therefore, only the rheological properties of mixed shell powder/SBS-composite-modified asphalt were studied.

**Figure 7:**
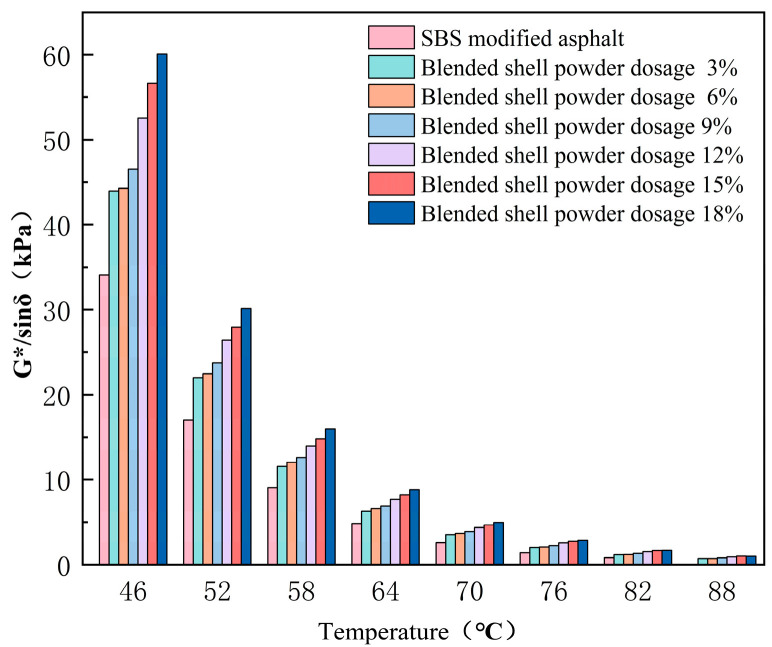
Rutting factor of SBS-modified asphalt with different proportions of mixed shell powder.

The rutting factor of SBS-modified asphalt changes with the content of mixed shell powder, as shown in Figure 7. The results of the temperature scanning test show that the addition of mixed shell powder enhances the ability of asphalt to resist flow deformation at high temperature, and the failure temperature grade of SBS-modified asphalt is increased from 82 °C to 88 °C.

**Figure 8:**
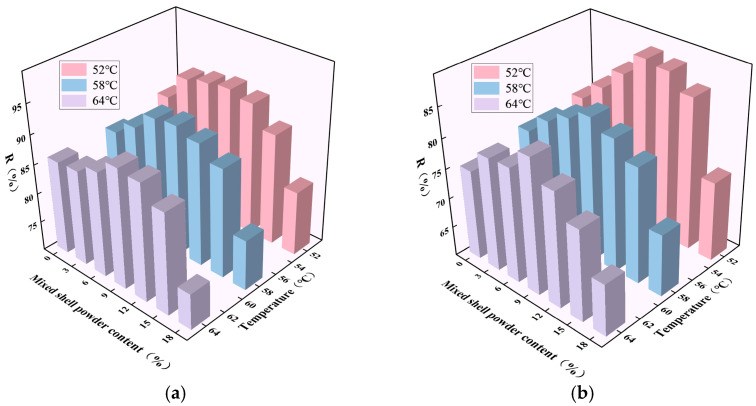
Strain recovery rate of SBS-modified asphalt with different proportions of mixed shell powder: (**a**) stress of 0.1 kPa; (**b**) stress of 3.2 kPa.

**Figure 9:**
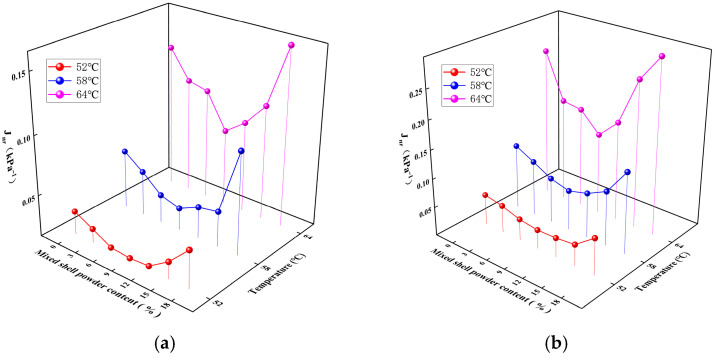
Unrecoverable creep compliance of SBS modified asphalt with different proportions of mixed shell powder: (**a**) stress of 0.1 kPa; (**b**) stress of 3.2 kPa.

The strain recovery rate R and unrecoverable creep compliance J_nr_ of SBS-modified asphalt with different mixed shell powder contents are shown in Figure 8 and Figure 9. Under the load of 0.1 kPa and 3.2 kPa, the effect of mixed shell powder content on the performance of SBS-modified asphalt showed a significant regular change. It is found that with the increase in the content of mixed shell powder, the strain recovery rate R of SBS-modified asphalt increases first and then decreases at the same temperature, and the corresponding irreversible creep compliance J_nr_ changes in the opposite direction. Both of them have inflection points when the content of mixed shell powder is 9%. When the content of mixed shell powder reaches 18%, the strain recovery rate of mixed shell powder/SBS composite modified asphalt is lower than that of SBS-modified asphalt, indicating that too high a content will weaken the elastic recovery ability of asphalt. Therefore, in the preparation of mixed shell powder/SBS-composite-modified asphalt, it is suggested that the content of mixed shell powder should be controlled within 15% to ensure that the modified asphalt has good deformation resistance and elastic recovery performance.

### 3.2. Performance Analysis of Mixed Shell Powder-Modified Asphalt Mixture

#### 3.2.1. High Temperature Stability

Asphalt mixture is prone to rutting and other deformation problems in a high-temperature environment, which seriously affects the service life of pavement and driving safety. Therefore, an in-depth study of its high-temperature stability is of great significance for optimizing pavement structure design and material selection.

**Figure 10:**
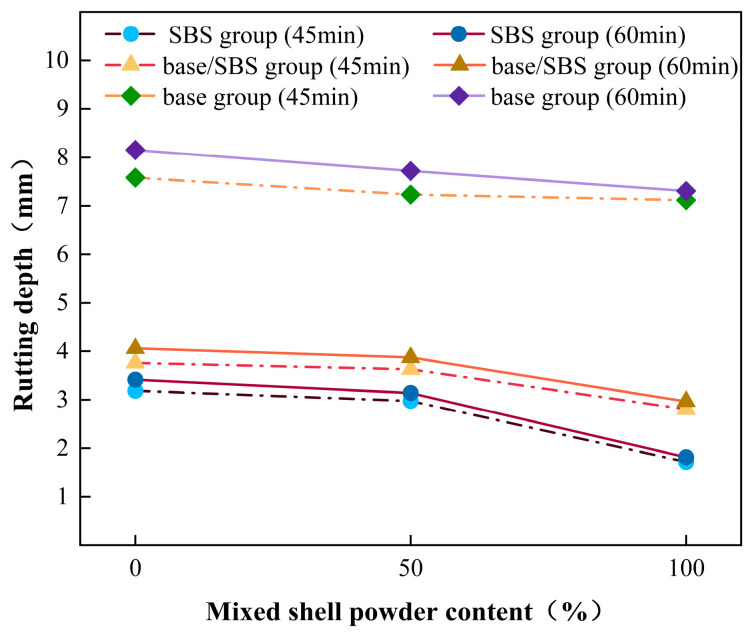
Rutting depth of asphalt mixtures with different blended shell powder contents.

To explore the influence of three different substitution amounts of mixed shell powder on the high-temperature stability of asphalt mixture, this experiment used mixed shell powder to replace limestone powder by 0%, 50%, and 100% in equal volume to prepare SBS-modified asphalt mixture, mixed shell powder/SBS composite-modified asphalt mixture, and base asphalt mixture. At the same time, the dynamic stability (DS) is used as the test index. The larger the index value is, the stronger the anti-rutting ability of the asphalt mixture is, and the better the high-temperature stability is. The results are shown in Figure 10 as the deformation after the rutting test. It can be seen from this that the final rutting depth of SBS-modified asphalt mixture, mixed shell powder/SBS composite-modified asphalt mixture, and base asphalt mixture decreases with the increase in shell powder substitution.

**Figure 11:**
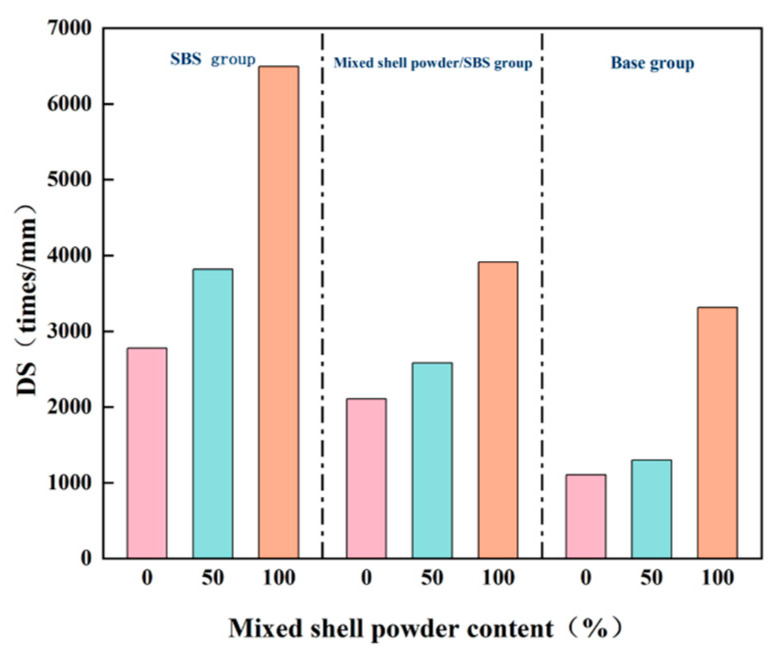
Dynamic stability of asphalt mixtures with different blended shell powder contents.

It can be seen from Figure 11 that the dynamic stability of the SBS-modified asphalt mixture is greater than 2400 times/mm, which meets the requirements of dynamic stability in the hot summer climate zone. The dynamic stability of the mixed shell powder/SBS composite-modified asphalt mixture. When the replacement amount of the mixed shell powder filler is 0%, the dynamic stability is greater than 2000 times/mm, which is suitable for the hot summer zone. When the replacement amount of mixed shell powder filler is 50% and 100%, the dynamic stability is greater than 2400 times/mm, which is suitable for the summer hot zone. The dynamic stability of the base asphalt mixture is greater than 800 times/mm, which meets the requirements of the current specification. In general, the use of mixed shell powder as a filler to replace limestone powder to prepare an asphalt mixture is helpful to improve the high-temperature stability of the asphalt mixture. With the increase in mixed shell powder content, the dynamic stability is continuously improved. When the mixed shell powder completely replaces the limestone powder filler, the deformation of the rut test block is the smallest, and the dynamic stability is the highest.

#### 3.2.2. Low Temperature Anti-Cracking Performance

**Figure 12:**
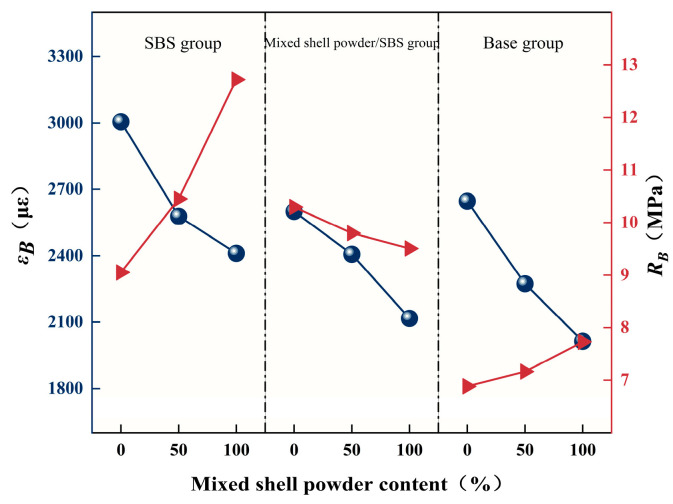
Low-temperature bending test results of asphalt mixtures with different blended shell powder contents.

In the evaluation of low-temperature performance, flexural tensile strength and failure strain are the key indicators. In general, the larger the values of these two indicators, the better the low-temperature crack resistance of the asphalt mixture. The low-temperature bending test results of the asphalt mixture with different mixed shell powder contents are shown in Figure 12, and the following specific analysis is carried out in combination with test data and specification requirements.

According to the specification, the failure strain value of the low-temperature bending test of the modified asphalt mixture should not be less than 2500 μm, and the failure strain value of the ordinary asphalt mixture should not be less than 2000 μm. According to the test results, in the SBS-modified asphalt mixture, when the replacement amount of mixed shell powder filler is 0% and 50%, the failure strain can meet the specification requirements of the modified asphalt mixture. The mixed shell powder/SBS composite modified asphalt mixture only meets the standard when the replacement amount of mixed shell powder filler is 0%. The strain values of the base asphalt mixture are greater than 2000 μm in the three cases of mixed shell powder substitution of 0%, 50%, and 100%, which meet the specification requirements of ordinary asphalt mixtures.

With the increase in the replacement amount of the mixed shell powder filler, the maximum bending strain of the specimen shows a decreasing trend, which indicates that the mixed shell powder filler will increase the material stiffness, which is not conducive to the low temperature stability of the asphalt mixture. This phenomenon is closely related to the physical properties of shell powder. Compared with limestone powder, shell powder has abundant pores and rough particle surfaces, and the adsorption capacity of asphalt is larger under the same volume. This characteristic can improve the cohesion of the SBS-modified asphalt mixture and the base asphalt mixture and then improve its flexural strength. However, at the same time, shell powder as a modifier will reduce the low-temperature ductility of asphalt, resulting in insufficient flexibility of the asphalt mixture. Therefore, under the same content of mixed shell powder filler, the maximum bending strain of mixed shell powder/SBS composite-modified asphalt mixture is smaller than that of the SBS-modified asphalt mixture. With the increase in the amount of the mixed shell powder filler, its strong adsorption effect will further increase the brittleness of the asphalt material, which will eventually lead to a decrease in the maximum flexural strain and flexural tensile strength of the mixed shell powder/SBS composite modified asphalt mixture.

#### 3.2.3. Water Stability

**Figure 13:**
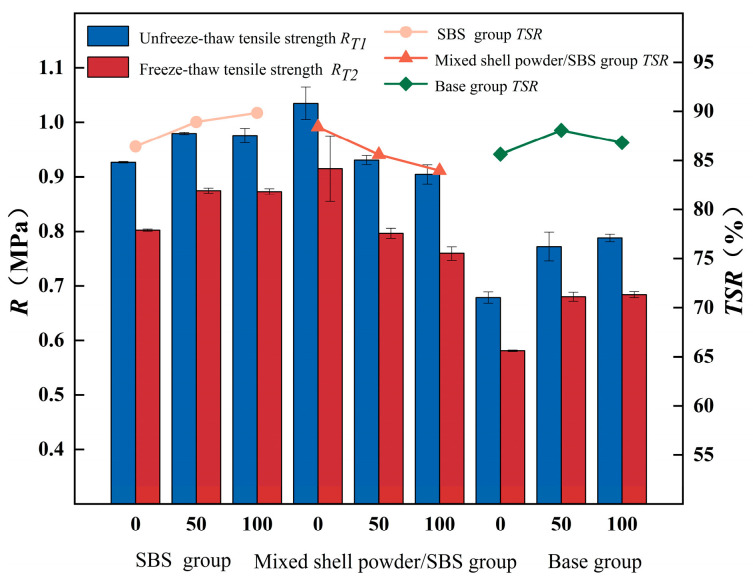
Freeze–thaw splitting test results of asphalt mixtures with different blended shell powder contents.

The water stability of the asphalt mixture is a key index to measure its ability to resist water erosion. It refers to the ability to resist the peeling of asphalt and aggregate and the damage to the mixture structure under the action of long-term water. The poor water stability will lead to a decrease in the strength of the asphalt mixture. Under the influence of external factors such as temperature change and vehicle load, the pavement is prone to cracking, which seriously affects its durability and performance. The tensile strength of the specimen was measured by the asphalt stability tester, and the tensile strength ratio was used as the evaluation index of water stability. The freeze–thaw splitting test was carried out. The test results under different mixed shell powder contents are shown in Figure 13.

It can be clearly observed that the tensile strength of the specimen after freeze–thaw cycle treatment is significantly lower than that of the non-freeze–thaw specimen, which indicates that the freeze–thaw effect will have a significant impact on the structural strength of the asphalt mixture. For base asphalt mixture and SBS-modified asphalt mixture, mixed shell powder can improve their splitting tensile strength and tensile strength ratio and help to improve water stability. This is because shell powder has rich pores and contains some organic components, which can be well combined with asphalt and are suitable as fillers for these two types of mixtures. However, for the mixed shell powder/SBS composite modified asphalt mixture, the mixed shell powder will weaken its water stability. In practice, limestone powder should be preferred as filler to ensure excellent water stability.

The adhesion of SBS-modified asphalt and mixed shell powder/SBS composite-modified asphalt to the surface of coarse aggregate was tested by the boiling method. It was found that the peeling area of the two kinds of asphalt was close to zero, the asphalt film did not move, and the adhesion grade was 5, which indicated that the adhesion between the two kinds of asphalt and aggregate was excellent. The water stability of mixed shell powder/SBS composite-modified asphalt is insufficient, which may be caused by the mixed shell powder filler. When mixed, shell powder is used as a filler; due to its many pores, high specific surface area, and mutual attraction between particles, it is prone to agglomeration. At the same time, the mixed shell powder/SBS composite-modified asphalt has small penetration, low ductility, and low light components, resulting in uneven dispersion of mixed shell powder in the mixture. This makes it easier for water to invade the interior of the mixture in the presence of water, which is not conducive to the water stability of the mixed shell powder/SBS composite-modified asphalt mixture.

## 4. Study on the Mechanism of Mixed Shell Powder-Modified Asphalt

### 4.1. Fourier Infrared Spectroscopy Test

In order to reveal the mechanism of action of mixed shell powder and asphalt at the chemical level and provide chemical theoretical support for the recommended dosage of mixed shell powder (9% in SBS-modified asphalt) proposed in this study, the samples of Fourier infrared spectroscopy tests were determined to be mixed shell powder, pure SBS-modified asphalt, and 9% mixed shell powder/SBS composite-modified asphalt.

**Figure 14:**
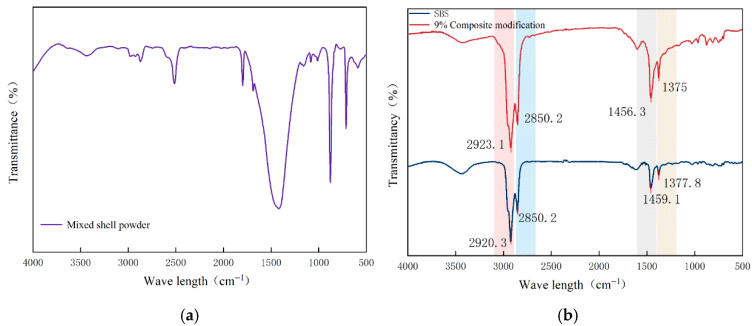
The infrared spectrum of SBS modified asphalt with different dosages of mixed shell powder modifier: (**a**) mixed shell powder; (**b**) SBS-modified asphalt.

As shown in Figure 14, the infrared spectrum of the mixed shell powder showed typical calcium carbonate mineral characteristics. A broad absorption peak appears in the range of 3000–4000 cm^−1^, which is the stretching vibration peak of the hydroxyl group, derived from the water molecules adsorbed on the surface of the shell powder. There are several strong absorption peaks in the range of 800–1500 cm^−1^, corresponding to the characteristic vibration of carbonate in calcium carbonate, which is the characteristic peak of shell powder. The overall peak shape is sharp, and the characteristics are clear, which is completely consistent with the conclusion that the main component of the shell powder is calcium carbonate. The spectrum of pure SBS-modified asphalt reflects the characteristics of organic polymer blending, and the key absorption peaks are clearly marked in the diagram. Additionally, 2923.1 cm^−1^ and 2850.2 cm^−1^ are the stretching vibration peaks of the saturated C-H bond, which mainly come from the saturated hydrocarbon components in asphalt and the alkane chain of the butadiene unit in SBS. The bending vibration peak at 1456.3 cm^−1^ is -CH_2_ bending vibration peak, and the symmetrical bending vibration peak at 1375 cm^−1^ is -CH_3_ bending vibration peak. These peak positions and strengths are in line with the typical chemical structure characteristics of SBS-modified asphalt. The spectral characteristics of 9% mixed shell powder/SBS composite-modified asphalt can clearly show the key rules by comparing with the spectrum of pure SBS-modified asphalt. From the peak position, 9% composite modified asphalt retains all the characteristic peaks of pure SBS modified asphalt without an obvious offset. From the peak shape and the presence or absence of peaks, no new characteristic peaks appeared, and no original characteristic peaks disappeared. This spectral feature fully shows that the sample spectrum only presents a simple superposition of the characteristic peaks of the two components of the mixed shell powder and the pure SBS-modified asphalt, and there is no formation, disappearance, or wavenumber shift in the functional groups. It is confirmed that the mixed shell powder and SBS-modified asphalt are only physically mixed, and no chemical reaction occurs. The slight change in the intensity of some peaks is only due to the change in the relative concentration of each component in the system after the mixing of the two components, which is a normal phenomenon in the physical mixing process. This change is also related to the physical adsorption of the porous structure of the mixed shell powder on the asphalt component. The porous structure of the shell powder adsorbs some of the asphalt and SBS components, resulting in a slight decrease in the concentration of the effective components in the system, which in turn causes a slight decrease in the intensity of the characteristic peaks.

In the performance test, the slight effect of mixed shell powder on the high-temperature stability and low-temperature ductility of SBS-modified asphalt can also be reasonably explained by the physical mixing mechanism. The physical filling and adsorption enhance the consistency and stiffness of the asphalt system, thus improving the high-temperature deformation resistance, while the rigid particle characteristics slightly weaken the flexibility of the asphalt. Based on this, if it is necessary to optimize the comprehensive performance of mixed shell powder-modified asphalt in the future, it is necessary to start with improving the efficiency of physical action, such as optimizing the dispersion of shell powder, and improving the bonding degree between particles and asphalt interface, rather than relying on the regulation of chemical action, which provides an important reference for the subsequent research direction.

### 4.2. Microscopic Morphology and Dispersion Characteristics

**Figure 15:**
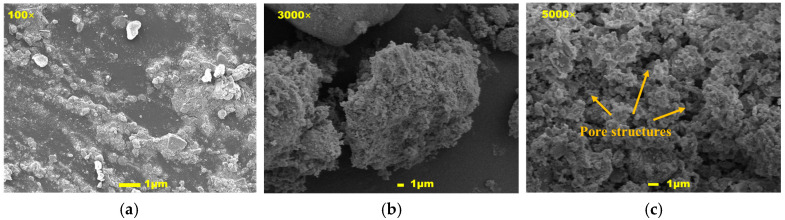
Scanning electron microscopy images of mixed shell powder: (**a**) 100 times; (**b**) 3000 times; (**c**) 5000 times.

The agglomeration problem is common in the modification of small-particle asphalt. The uniform dispersion of the modifier in asphalt is the premise to give full play to its performance. In Figure 15, scanning electron microscope observation of pure mixed shell powder showed that the particles were irregularly blocky as a whole, and the surface was rough and gully. Under high-magnification observation, the internal calcium carbonate crystals were interwoven in the form of needles, and a large number of pore structures with uneven pore sizes were formed in the crystal gaps.

**Figure 16:**
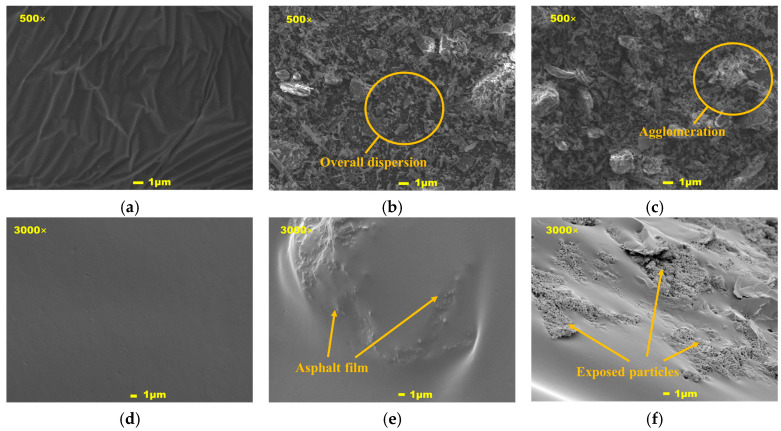
Scanning electron microscopy images: (**a**) SBS-modified asphalt at 500 times; (**b**) 9% mixed shell powder at 500 times; (**c**) 18% mixed shell powder at 500 times; (**d**) SBS-modified asphalt at 3000 times; (**e**) 9% mixed shell powder at 3000 times; (**f**) 18% mixed shell powder at 3000 times.

The SBS-modified asphalt, 9% and 18% mixed shell powder/SBS composite-modified asphalt, and 500 times and 3000 times microscopic images are shown in Figure 16. When the mixed shell powder is not added, the SBS-modified asphalt matrix is in a uniform and continuous colloidal state, without obvious particle impurities or voids. After adding 9% mixed shell powder, the particles showed local aggregation but overall dispersion. Most of them were distributed in the form of a single or a small number of small aggregates, and there was no large area of agglomeration. The surface of the particles was completely wrapped by a continuous asphalt film, and the interface was tightly bonded without debonding. In this dispersed state, the porous structure of shell powder can fully adsorb the light components of asphalt, forming a composite structure of rigid particles and an asphalt adsorption layer. When the content is increased to 18%, a large number of shell powder particles form large-sized aggregates. The particles in the aggregates squeeze each other, and the internal particles are obviously exposed. The asphalt matrix is discontinuous due to excessive particle filling, and local microcracks are generated. The formation of aggregates is due to the increase in surface energy between particles at high content, and the electrostatic adsorption exceeds the dispersion ability of the asphalt matrix. Exposed particles and microcracks will lead to stress concentration, reduce elastic recovery performance, and aggravate the risk of brittle fracture at low temperatures. This also verifies the conclusion that the content of mixed shell powder should not exceed 15% at the micro level.

Combined with the observation results of scanning electron microscopy and the previous macroscopic performance test data, a clear correlation mechanism can be established. The porous rough structure of the mixed shell powder adsorbs the light components of asphalt to form a rigid composite structure by increasing the specific surface area and mechanical occlusion, which improves the consistency and deformation resistance of asphalt. The macroscopic performance is the increase in softening point and rutting factor. When the content is low, the shell powder is uniformly dispersed, the damage to the continuity of the asphalt is small, and the low-temperature ductility is limited. When the content is high, the agglomeration of particles leads to the discontinuity of the asphalt matrix, which causes stress concentration, enhances the brittleness at low temperature, and decreases the ductility significantly. The integrity of the asphalt film on the surface of the shell powder determines the interfacial bonding strength. The asphalt film is continuous, and the interface is tightly bonded at 9%. Under 18% dosage, some particles are exposed, the interface bonding is weak, and it is easy to become a performance short board. In summary, the scanning electron microscope test verifies the mechanism of action of mixed shell powder on SBS-modified asphalt from the micro level and clarifies that the 9% content is the best interval for both dispersion uniformity and performance optimization, providing direct micro-evidence for the previously recommended content. At the same time, it points out the path for the subsequent optimization direction, such as reducing agglomeration through surface modification, optimizing the mixing process, and selecting an appropriate dosage.

## 5. Conclusions

In this study, through material preparation performance tests and microscopic mechanism analysis, the influence of mixed shell powder on the road performance of base asphalt, SBS-modified asphalt, and the corresponding mixture was systematically explored, and the action law and applicable dosage were clarified. The main conclusions are as follows:

(1) With the increase in mixed shell powder content, the penetration of base asphalt and SBS-modified asphalt continued to decrease, and the softening point increased first and then decreased. The ductility of base asphalt with mixed shell powder content greater than 3% is unable to meet the specification, while SBS-modified asphalt handles up to 9% with only a slight decrease. The viscosity at 135 °C increases with the increase in the content. Considering the high temperature stability and construction adaptability, the recommended content of mixed shell powder in SBS-modified asphalt is 9%.

(2) The dynamic stability of the mixed shell powder/SBS composite modified mixture with 50% and 100% substitution amounts is more than 2400 times/mm, meeting the requirements of the summer hot zone. Increasing the substitution amount of mixed shell powder reduces low-temperature crack resistance. SBS modified mixtures meet the 2500 μm strain limit when the substitution amount is 0% or 50%, while base mixtures remain above the 2000 μm standard at all substitution levels. Replacing 50–100% of the filler with mixed shell powder can improve the water stability of the base and single SBS-modified mixture, but it will reduce the performance of the composite-modified mixture.

(3) Fourier infrared spectroscopy showed that the mixed shell powder and SBS-modified asphalt were only physically mixed, no new functional groups were generated, and only characteristic peaks were superimposed. The peak intensity change was due to the physical adsorption of the porous structure of the shell powder on the asphalt component; scanning electron microscopy showed that the shell powder particles were uniformly dispersed and the interface was tightly bonded when the content was 9%. When the content was 18%, agglomeration occurred and caused microcracks in the asphalt matrix. The difference in microscopic morphology directly led to macroscopic performance, low content optimization performance, high content weakening elastic recovery, and low temperature performance.

## Figures and Tables

**Table 1:** Technical indicators of materials.

Materials	Test Items	Specification Requirement	Measured Value
Asphalt	Needle penetration of base asphalt (25 °C)/0.1 mm	60~80	75.8
	Softening point of base asphalt/℃	≥45	47.9
	Ductility of base asphalt (10 °C, 5 cm/min)/cm	≥15	36.1
	Needle penetration of SBS modified asphalt (25 °C)/0.1 mm	60~80	64.10
	Softening point of SBS modified asphalt/℃	≥55	77.7
	Ductility of SBS-modified asphalt (5 °C, 5 cm/min)/cm	≥30	58.6
Coarse aggregate	Crushing value/%	≤26	20.1
	Los Angeles wear value/%	≤28	18.2
	Apparent relative density	≥2.6	2.714
	Relative density of hair volume	-	2.668
	Water absorption/%	≤2.0	0.64
Fine aggregate	Apparent relative density	≥2.5	2.796
	Firmness/%	≥12	Compliant
	Mud content/%	≤3	1.2
Limestone filler	Apparent density/g·cm^−3^	≥2.5	2.63
	Water content/%	≤1	0.42
	Appearance	No agglomerates	No agglomerates
	Hydrophilic coefficient	<1	0.64
	Plasticity index	<4	3.42

## Data Availability

The original contributions presented in this study are included in the article. Further inquiries can be directed to the corresponding authors.
